# Poststroke Muscle Architectural Parameters of the Tibialis Anterior and the Potential Implications for Rehabilitation of Foot Drop

**DOI:** 10.1155/2014/948475

**Published:** 2014-07-16

**Authors:** John W. Ramsay, Molly A. Wessel, Thomas S. Buchanan, Jill S. Higginson

**Affiliations:** ^1^Biomechanics and Movement Science Program, University of Delaware, Newark, DE 19716, USA; ^2^Delaware Rehabilitation Institute, University of Delaware, Newark, DE 19713, USA; ^3^Department of Biomedical Engineering, University of Delaware, Newark, DE 19716, USA

## Abstract

Poststroke dorsiflexor weakness and paretic limb foot drop increase the risk of stumbling and falling and decrease overall functional mobility. It is of interest whether dorsiflexor muscle weakness is primarily neurological in origin or whether morphological differences also contribute to the impairment. Ten poststroke hemiparetic individuals were imaged bilaterally using noninvasive medical imaging techniques. Magnetic resonance imaging was used to identify changes in tibialis anterior muscle volume and muscle belly length. Ultrasonography was used to measure fascicle length and pennation angle in a neutral position. We found no clinically meaningful bilateral differences in any architectural parameter across all subjects, which indicates that these subjects have the muscular capacity to dorsiflex their foot. Therefore, poststroke dorsiflexor weakness is primarily neural in origin and likely due to muscle activation failure or increased spasticity of the plantar flexors. The current finding suggests that electrical stimulation methods or additional neuromuscular retraining may be more beneficial than targeting muscle strength (i.e., increasing muscle mass).

## 1. Introduction

In the United States alone, about 795,000 people suffer from a new or recurrent stroke each year [1]. Stroke survivors often suffer from hemiparesis or muscle weakness on one side of the body. Foot drop commonly occurs from muscle weakness in the paretic leg and manifests itself as a decrease in dorsiflexion range of motion [2]. For many poststroke survivors, paretic limb foot drop increases the risk of stumbling and falling and decreases functional mobility [2]. It is unclear whether dorsiflexor weakness is solely due to neurological impairment following stroke or whether changes in the muscle architecture are additional contributing factors. Muscle fascicle length and pennation angle (i.e., the angle in which the fascicles insert themselves into the aponeuroses of the muscle) are two architectural parameters that can influence how a muscle generates force. Varying these two parameters can alter the functional ability of a muscle, including range of motion and total force production [3]. Therefore, changes in fascicle length or pennation angle may contribute to post-stroke dorsiflexor weakness [4].

Medical imaging techniques (e.g., ultrasonography and magnetic resonance imaging) are often used to study muscle architecture* in vivo* in healthy populations and patients with neurological disorders [5–11]. However, little is known about poststroke muscle architectural changes. In the upper extremity, shorter fascicle lengths have been reported for the brachialis muscle on the affected side using ultrasound [8]. Similarly, Gao et al. [4] reported a decrease in paretic medial gastrocnemius muscle fascicle length, pennation angle, and range of motion as compared to healthy adults. Additionally, Ramsay et al. [9] observed no differences between paretic and nonparetic muscle volumes for the tibialis anterior (TA) [9]. However, changes in other architectural parameters such as fascicle length, pennation angle, and overall muscle length may also contribute to poststroke dorsiflexor weakness. A better understanding of hemiparetic TA muscle architecture will allow clinicians to apply different techniques to help patients to reduce foot drop, thus improving walking mobility.

The objective of this study was to use medical imaging techniques to quantify the architectural changes that occur between paretic and nonparetic tibialis anterior muscles. Muscle parameters we measured were muscle volume, muscle length, muscle fascicle length, and pennation angle. We hypothesized that there would be no changes between the paretic and nonparetic side for any muscle parameter, which would indicate that dorsiflexor weakness is primarily a neurological impairment.

## 2. Methods

### 2.1. Subjects

Ten poststroke subjects (61 ± 10 yrs., 8 males, 52 ± 40 months since stroke) were recruited for this study (Table 1). Subjects had no other conditions that affected walking (e.g., Parkinson's disease and joint replacement), no bone or joint problems in the legs or spine, or no shortness of breath without exertion in the last 6 months, could understand spoken instructions and were able to communicate with the investigators, had no implanted magnetic or electronic device, and could walk without the assistance of another person but may have used a leg brace or an assistive device if needed. All subjects were provided and signed an informed consent approved by the University of Delaware review board.

### 2.2. Magnetic Resonance Imaging Protocol

Muscle volumes and muscle belly length for the tibialis anterior muscle were obtained from magnetic resonance images. Muscle volumes were calculated using methods described previously by Ramsay et al. [9]. Axial T1-weighted MR images were acquired for both legs using a 1.5 T Signa LX scanner (GE Medical, Milwaukee, WI). To limit any movement during scanning and maintain neutral hip rotation, subjects lay supine with their feet taped together at the toes. Two overlapping regions of the lower leg were imaged using a repetition time of 450 ms, echo time of 10 ms, slice thickness of 10 mm, and a distance between slices of 11.5 mm. IMOD software (University of Colorado, Boulder, CO; Kremer et al. [12]) was used to manually trace the boundary of the TA muscle over the entire muscle length. Cross-sectional areas were calculated using a trapezoidal integration algorithm and, after adjusting the pixel threshold for fat suppression, volume was calculated by summing the cross-sectional areas multiplied by the slice thickness over the length of the muscle. Other studies have used similar gray-level (pixel intensity) methods to distinguish between lean skeletal muscle and adipose tissue [13–15]. Using muscle belly origin and insertions, muscle length (*L* ^m^) was calculated by multiplying the space between slices (11.5 mm) by the number of scan slices spanning each muscle. Mean values for volume and muscle length were calculated for the paretic and nonparetic sides.

### 2.3. Ultrasound Protocol

**Figure 1:**
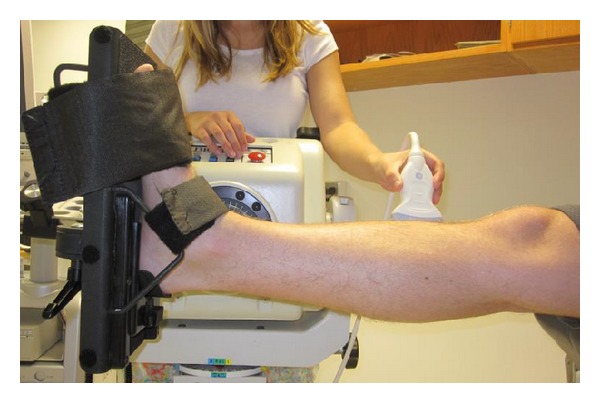
Subject in isometric dynamometer with ankle secured in neutral position. Ultrasound images were taken at the midbelly of the tibialis anterior. Care was taken to keep the probe perpendicular to the skin. Enough pressure was applied to maintain proper contact between the probe and skin without significantly deforming the muscle.

**Figure 2:**
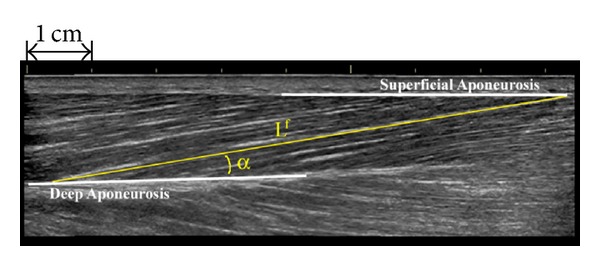
Paretic tibialis anterior fascicle (*L* ^f^) and pennation angle (*α*) for one subject using extended field-of-view.

Subjects were seated upright in an isometric dynamometer (Biodex, Shirley, NY) with their knee fully extended and ankle secured in neutral posture (Figure 1). Individual paretic and nonparetic muscle fascicle lengths (*L* ^f^) and pennation angles (*α*) were obtained from a GE LOGIQ P6 (GE Healthcare, Waukesha, WI, USA) ultrasound device. Longitudinal ultrasound images of the TA were collected using a B-mode scanner with a 15 MHz high-resolution linear array probe (ML6-15). For all subjects, two images of both legs were acquired at neutral ankle position (Figure 2). All images were taken with the subject in a resting condition at each joint angle for both legs. Extended field-of-view was used when fascicle length was longer than the standard field-of-view. This method has been shown to be accurate within 5% of a measured value [16]. Fascicle length was measured from the deep aponeurosis to the superficial aponeurosis (Figure 2). Pennation angle was measured from the muscle fascicle to the deep aponeurosis (Figure 2). Fascicle length was normalized to the subject's height and both normalized fascicle length and pennation angle were averaged across repeat scans.

### 2.4. Statistical Analysis

Muscle volume, muscle length, normalized fascicle length, and pennation angle at neutral joint angle were determined to be normally distributed data using Lilliefors test and were compared between limbs using paired *t*-tests. The average absolute difference between paretic and nonparetic muscle belly lengths was also determined across all subjects.

## 3. Results

### 3.1. Muscle Volume and Muscle Length

There were no statistical differences (*P* = 0.934) between the paretic (111.5 ± 34.8 cm^3^) and nonparetic (112.2 ± 25.8 cm^3^) muscle volumes (Table 2). Muscle lengths for the paretic and nonparetic sides were not statistically different (*P* = 0.659) and averaged 28.9 ± 2.0 cm and 28.6 ± 2.9 cm, respectively (Table 2). The average difference between paretic and nonparetic muscle length was 0.4 cm.

### 3.2. Fascicle Length and Pennation Angle at Neutral Angle

Normalized fascicle lengths at neutral ankle angle were not significantly different (*P* = 0.663) between paretic (0.150 ± 0.024) and nonparetic (0.148 ± 0.025) sides. Paretic pennation angles (13.4 ± 2.7°) were not significantly different (*P* = 0.069) than nonparetic pennation angles (10.9 ± 1.9°) at neutral ankle angle (Table 2).

## 4. Discussion

In the study, we used noninvasive medical imaging techniques (i.e., MRI and ultrasound) to quantify changes between the paretic and nonparetic tibialis anterior muscle in poststroke individuals. Muscle volumes, muscle lengths, fascicle lengths, and pennation angles at neutral joint angle were all similar between sides. Overall, we determined that there are no meaningful bilateral differences in poststroke tibialis anterior muscle architecture. This indicates that these subjects have the muscular capacity to dorsiflex their foot and that dorsiflexor strength is primarily inhibited by neurological impairment.

Muscle atrophy has been shown to exist after stroke, yet most look at cross-sectional area [17] or volume [9, 11, 18, 19] as a measure of muscle size. However, it has been suggested that plantar flexor muscle length shortens in chronic stroke survivors due to the shortening and stiffening of plantar flexor muscle fibers [20]. To determine whether this mechanism also occurs in the TA and give a more representative view on poststroke atrophy of the TA, we presented both muscle volume and muscle length data. Mean muscle volumes of these subjects were similar to those found in our previous work and confirm that muscle volumes are not changing between the paretic and nonparetic limbs. Additionally, muscle lengths were also similar between sides. Together these results confirm that the size of the poststroke TA is not changing following stroke.

While overall size changes may not occur after stroke, we were also interested in whether any fascicular changes occurred both at neutral position and across each individual's range of motion. Changes at the fascicular level will also influence the ability of a muscle to generate force since muscle force is transmitted to the tendon along the muscle fascicle axis and is proportional to the cosine of the pennation angle. Poststroke medial gastrocnemius fascicle lengths have been reported to be shorter and pennation angles smaller than healthy values [4], a combination that suggested that the passive fascicular tension increased. Additionally, an increase in pennation angle has been reported to coincide with muscle hypertrophy [21]. However, we found no results that confirmed any fascicular changes in the tibialis anterior. In neutral ankle position, there were no differences in fascicle length and pennation angle between sides.

The overall implication for rehabilitation of foot drop provided by our findings is that interventions that are directly designed to target dorsiflexor weakness should be designed to address the underlying physiological deficit, which is neurological impairment (e.g., decrease in voluntary muscle activation, the ability to activate higher numbers of motor units, or number of active motor units). Rehabilitation targeting muscle strength solely by increasing muscle mass will be ineffective, as atrophy and architectural differences do not exist in the TA. One useful intervention technique is electrical stimulation. Electrical stimulation of the dorsiflexors has been used to improve walking in subjects with hemiparesis [22, 23] and can be implanted for prolonged, daily use [23]. Other neurological benefits from dorsiflexor electrical stimulation are that it may decrease plantar flexor spasticity through reciprocal inhibition [24–26] and also decrease the plantar flexor stretch reflex [27, 28]. However, while electrical stimulation is useful as both an orthosis and rehabilitation tool, it is not common clinical practice [28]. In light of our findings, we suggest that use of electrical stimulation or an additional form of neuromuscular retraining would be beneficial because it addresses the neurological deficits contributing to dorsiflexor weakness.

There are a few assumptions and limitations worth noting when interpreting these results. First, we assumed that the tibialis anterior muscle is the only dorsiflexor muscle related to foot drop. We believe that including the deeper muscles would not change the outcome of this study as the tibialis anterior is the largest of dorsiflexor muscles and likely has the largest effect on movement. Secondly, we recognize that the muscle parameters were measured during a resting condition and that changes would occur during muscle contraction. We also recognize that overall muscle architecture is not completely described in the work. Muscle fascicles are comprised of muscle fibers and, on a smaller scale, a series of sarcomeres. Techniques using laser diffraction [29] have been able to measure such parameters and may be of interest to fully describe poststroke tibialis anterior muscle architecture. It is also important to note that the parameters we chose are specific to active muscle force production, not passive. Achilles tendon properties have been shown to change between the paretic and nonparetic sides [20], and it is still unclear whether the properties of the tibialis anterior tendon change following stroke. Finally, we did not look at changes in muscle fiber type although [30] performed histological biopsies of the tibialis anterior and found that type II muscle fibers reduce in number and diameter, with a predominant shift towards type I fibers. This change in fiber type ratio, if consistent for chronic stroke survivors, may be indicative of activation failure or disuse.

## 5. Conclusion

We found no clinically meaningful differences in any architectural parameter between the paretic and nonparetic tibialis anterior muscles. Therefore, we assert that paretic dorsiflexor weakness is not due to changes in muscle architecture but more likely due to activation failure or increased spasticity in plantar flexors. Since these individuals have the muscular capacity to dorsiflex their foot, foot drop must be due to neurological impairments (e.g., decrease in voluntary muscle activation, the ability to activate higher numbers of motor units, or number of active motor units). Electrical stimulation may be an effective rehabilitation tool to address these factors and should be considered more often in the clinical setting.

## Figures and Tables

**Table 1:** Subject demographics for 10 poststroke individuals.

Subject number	Gender	Side of paresis	Age	Months since stroke	Mass (kg)	Height (m)	FM_LE (out of 34)	Presence of clonus
1	M	R	65	89	73.1	1.727	23	Sustained
2	M	R	76	80	87.2	1.867	12	None
3	M	R	62	12	77.8	1.740	13	Present∗
4	M	R	51	9	69.5	1.803	15	Present∗
5	F	L	74	10	93.7	1.626	19	None
6	M	L	59	85	102.0	1.803	26	Present∗
7	M	R	63	12	74.9	1.803	25	None
8	M	L	46	23	99.1	1.740	23	None
9	F	R	48	105	83.9	1.702	16	None
10	M	L	69	99	102.3	1.778	22	None

*Clonus was present but minimal.

**Table 2:** Muscle parameter data and test statistics for the poststroke tibialis anterior muscle.

	Paretic	Nonparetic	Statistic
Muscle volume (cm^3^)	111.5 ± 34.8	112.2 ± 25.8	*P* = 0.934

Muscle length (cm)	28.9	28.6	*P* = 0.659

Neutral ankle normalized fascicle length (cm)	0.150	0.148	*P* = 0.663

Neutral ankle pennation angle (°)	13.4	10.9	*P* = 0.069
